# Toward a model of repeat-driven chromosomal fragility in Triticeae: an integrated structural omics perspective

**DOI:** 10.1186/s12864-026-12963-w

**Published:** 2026-05-26

**Authors:** Michał T. Kwiatek

**Affiliations:** Plant Breeding and Acclimatization Institute - National Research Institute in Radzików, Błonie, Poland

**Keywords:** Structural genomics, Pangenomics, Genome instability, Repetitive DNA, Chromosomal rearrangements, Triticeae, 3D chromatin, Omics-based breeding

## Abstract

**Background:**

Repetitive DNA dominates cereals genomes and plays a major yet poorly understood role in shaping structural genome variation. Recent advances in long-read sequencing, pangenomics, and 3D chromatin mapping have revealed that chromosomal rearrangements in Triticeae frequently arise within repeat-rich heterochromatin. However, the molecular and structural omics determinants underlying this recurrent fragility remain largely unresolved.

**Main body:**

This review synthesizes insights from pangenome analyses, cytogenetics, long-read genome assemblies, Hi-C–based 3D chromatin architecture, and meiotic double-strand break (DSB) mapping to outline a unified Repeat-Driven Chromosomal Fragility (RDCF) model. It shows that specific repetitive DNA architectures, short sequence motifs, and chromatin environments create Repetitive Fragility Zones (RFZs) that predispose chromosomes to double strand breaks and non-allelic repair. Comparative omics evidence from Triticeae species indicates that RFZs are evolutionarily conserved, recur across lineages, and are enriched in structural variants identified by high-throughput sequencing. Integrating these multi-omics datasets highlights how repeat-driven fragility shapes genome plasticity, introgression dynamics, and polyploid evolution in cereals.

**Conclusion:**

The RDCF framework connects structural omics signatures with chromosomal behavior and provides a conceptual basis for predictive fragility maps that may improve chromosome engineering, targeted introgression, and genome stabilization in crop breeding. The evidence presented here supports the view that repetitive DNA is not merely a passive component of large genomes but an active driver of structural and evolutionary innovation in Triticeae. Strengthening multi-omics integration will be essential for translating these insights into tools for crop improvement.

## Introduction

Structural rearrangements in eukaryotic genomes arise through diverse mutational mechanisms associated with DNA recombination, replication, and repair. These rearrangements tend to occur non-randomly, preferentially within genomic regions whose architecture predisposes them to instability. Structural variants typically originate when DSBs or replication forks are improperly repaired, giving rise to deletions, duplications, inversions, or translocations. The chromosomal distribution of breakpoints reflects intrinsic features of genome organization, as they frequently cluster within unstable regions such as Low Copy Repeats (LCRs), repetitive elements, or non-canonical (non-B) DNA structures that impede replication fork progression [1]. These loci often act as genomic “hotspots” of fragility and may serve as substrates for recombination- or repair-mediated rearrangements that reshape chromosome structure and function.

In eukaryotes, repetitive sequences constitute the principal drivers of such fragility. They can facilitate illegitimate recombination, alter replication dynamics, and affect the probability that particular DNA repair pathways are engaged [2]. Studies in mammals have demonstrated that repeat-rich loci often coincide with regions prone to non-allelic homologous recombination (NAHR), non-homologous end joining (NHEJ), or replication-based mechanisms such as Fork Stalling and Template Switching (FoSTeS) and Microhomology-Mediated Break-Induced Replication (MMBIR), directly linking genome architecture to rearrangement formation [3, 4].

Repetitive DNA dominates the genomes of Triticeae, accounting for over 80% of the total nuclear DNA in *Triticum aestivum* and its relatives [5, 6]. Organized into complex arrays of tandem and dispersed elements, these repeats form the structural foundation of heterochromatin and play essential roles in chromosome identity, pairing, and evolution. Repetitive sequences act as dynamic elements that can both stabilize and destabilize chromosomal architecture, depending on their organization and genomic context [7]. In Triticeae, the extreme abundance of repeats, together with extensive segmental duplications and polyploidy, creates a genomic environment inherently prone to breakage and rearrangement.

Although repeat-mediated rearrangements have been extensively studied in animal systems, their molecular basis in plants remains poorly understood. The large, repeat-dense genomes of Triticeae provide an exceptional model for investigating how repetitive DNA contributes to chromosomal instability, repair pathway selection, and karyotype evolution. Evidence from other eukaryotes suggests that repetitive and structurally complex regions act as preferential sites of DSB formation, replication fork stalling, and subsequent rearrangement; however, the extent and mechanisms of these processes in Triticeae are yet to be fully elucidated.

The Triticeae repeatome encompasses a wide diversity of repetitive DNA families, dominated by long terminal repeat (LTR) retrotransposons of the *Ty1/Copia* and *Ty3/Gypsy* superfamilies, as well as tandem repeats, microsatellites, and satellite DNA [8, 9]. In bread wheat, retrotransposons constitute more than 65% of the genome, while tandem repeats account for approximately 5–10%. Cytogenetically, tandemly arranged sequences such as pTa-86, pTa-535, pTa-713, pSc119.2, and pAs1 serve as robust chromosome markers and are widely used to trace chromosomal rearrangements in hybrids and introgression lines (Table 1) [10–12].

**Table 1:** Representative repetitive DNA sequences associated with chromosomal rearrangements in Triticeae

Sequence	Repeat type	Approximate monomer length	Genomic abundance	Typical chromosomal distribution	Cytogenetic relevance	References
pTa-86	Tandem satellite repeat	~ 86 bp	Moderate to high in Triticeae genomes	Subtelomeric and interstitial heterochromatin in wheat and related species	Frequently used FISH marker for identifying chromosome segments involved in rearrangements	[10–13]
pTa-535	Tandem satellite repeat	~ 340 bp	Moderate	Subtelomeric regions of wheat chromosomes	Cytogenetic probe for detecting structural polymorphisms and introgressions	[10–12]
pTa-713	Tandem satellite repeat	~ 120–130 bp	Moderate	Subtelomeric and interstitial chromosomal regions	Often co-localizes with other tandem arrays forming repeat blocks	[10–12]
pSc119.2	Tandem repetitive sequence from rye genome	~ 120 bp	High in *Secale*; present in wheat–rye introgression lines	Subtelomeric regions of rye chromosomes and wheat–rye translocations	Key probe for identifying rye chromatin in triticale and wheat–rye hybrids	[11, 14, 15]
pAs1	Dispersed repetitive sequence related to retrotransposons	Variable (~ 200–300 bp probe fragments)	High in wheat D genome	Interstitial regions of chromosomes, particularly in the D genome	Widely used marker for chromosome identification and detection of rearrangements	[11, 15, 16]
Cereba	Centromere-associated LTR retrotransposon	~ 7–8 kb	Highly abundant in centromeric regions	Centromeric chromatin of Triticeae chromosomes	Major component of cereal centromeres associated with rearrangement hotspots	[17–21]
Quinta	Gypsy-type retrotransposon	~ 8–10 kb	High in pericentromeric regions	Pericentromeric heterochromatin in wheat and *Aegilops*	Frequently enriched near chromosomal breakpoint regions	[8, 19, 22]

An important functional fraction of Triticeae repetitive DNA is concentrated in centromeric and pericentromeric regions. Plant centromeres are typically composed of large arrays of satellite repeats interspersed with centromere-associated retrotransposons, forming specialized chromatin domains responsible for kinetochore assembly and chromosome segregation. In cereals, these domains are frequently enriched in Ty3/Gypsy-derived retrotransposons such as the *Cereba* family together with tandem satellite sequences that define centromeric chromatin organization [17, 18]. The identity of functional centromeres is determined epigenetically by the incorporation of the centromere-specific histone variant CENH3, which nucleates kinetochore formation and ensures proper chromosome segregation during mitosis and meiosis.

Recent chromosome-scale and telomere-to-telomere genome assemblies have shown that plant centromeres are highly dynamic regions characterized by rapid turnover of satellite arrays and retrotransposon insertions. Comparative studies in cereals and model plants such as *Arabidopsis thaliana* demonstrate that centromeric repeats interact with specific epigenetic modifications and chromatin states that stabilize kinetochore domains while simultaneously allowing structural evolution of centromeric DNA [23]. These repeat-rich centromeric environments therefore represent specialized genomic compartments where repetitive DNA contributes both to chromosome stability and to long-term genome evolution.

Comparative FISH mapping across *Aegilops*, *Hordeum, Secale* and *Triticum*, species demonstrates substantial variation in the number, size, and chromosomal distribution of repetitive loci [14–16, 24, 25]. For example, the A genome of bread wheat typically shows lower repeat density and higher gene content compared with the more heterochromatic B and D genomes [8, 26–28]. This uneven repeat landscape influences chromatin compaction, replication timing, and the formation of subchromosomal “repeat blocks” that delineate evolutionary rearrangements. In many Triticeae species, satellite arrays occupy subtelomeric and pericentromeric regions, functioning both as structural anchors and as hotspots of variation [19, 29].

Polyploidization, a defining feature of Triticeae evolution, has been accompanied by pronounced remodeling of the repetitive fraction. Following hybridization, newly formed polyploids undergo “genome shock,” characterized by transposon activation, satellite DNA copy-number changes, and heterochromatin reorganization [30]. Comparative analyses of diploid, tetraploid, and hexaploid wheats reveal both amplification and elimination of specific repeat families, with centromeric and pericentromeric sequences being particularly dynamic [31–36]. Studies in *Brachypodium* polyploids [37–40] further demonstrate that centromeric tandem repeats and associated LTR retrotransposons undergo structural rearrangements during polyploidization, processes likely mirrored in Triticeae. Recent reconstructions of ancestral Triticeae and wheat lineage genomes [19, 41] have provided a phylogenomic framework for tracing rearrangements across diploid and polyploid species, revealing both conserved and lineage-specific translocation patterns.

At the molecular level, genome remodeling involves a balance between sequence turnover and chromatin reprogramming. Amplification of LTR retrotransposons during hybridization can induce local heterochromatin expansion, whereas repeat loss through unequal crossing-over or recombination-mediated deletion contributes to genome downsizing [42]. The equilibrium between amplification and elimination determines genome size stability and generates structurally variable regions that are predisposed to breakage [43–47].

The advent of high-quality pangenomes for wheat and related species has transformed the understanding of repeat-driven genome plasticity. The Wheat Pangenome Project [5] and subsequent multi-assembly analyses [8, 27] revealed pervasive structural variants, including deletions, duplications, inversions, and translocations, concentrated in repeat-rich regions. High-resolution alignments suggest that many structural variant breakpoints coincide with retrotransposon clusters or satellite arrays, supporting the concept that repetitive DNA predisposes genomic loci to DSB formation and illegitimate repair [9, 48–51].

Long-read sequencing technologies (ONT, PacBio HiFi) now enable precise characterization of these complex repeat architectures. Comparative analyses across 17 wheat cultivars [27] have shown that local repeat density correlates with GC content, recombination suppression, and the occurrence of structural rearrangements. In some cases, nearly identical repeat blocks occur in non-syntenic positions, indicative of historical ectopic recombination or translocation events. These findings corroborate cytogenetic observations that repetitive sequences demarcate chromosomal regions prone to structural change.

Collectively, the Triticeae repeat landscape forms a mosaic of chromosomal domains differing in stability. Euchromatic, gene-rich regions are relatively stable, whereas heterochromatic, repeat-dense regions exhibit high plasticity and harbor recurrent breakpoints. The accumulation of specific short motifs within these domains, such as those identified in triticale chromosmome breakpoints [13], may further influence DNA secondary structure and susceptibility to replication stress, generating local “micro-hotspots” of breakage. These RFZs likely represent the structural link between repetitive DNA organization and the cytologically observed chromosomal instability characteristic of Triticeae hybrids and polyploids. Overall, the repetitive DNA landscape of Triticeae is both architecturally complex and dynamically unstable. It provides not only the physical substrate for heterochromatin formation but also a plausible structural basis for chromosomal fragility. Elucidating how specific repeat motifs, densities, and chromatin contexts interact remains essential to understanding why certain chromosomal regions recurrently undergo breakage and translocation during evolution and hybridization. Despite progress in cytogenetic mapping and genome assembly, the molecular determinants of chromosomal fragility in plants remain largely unresolved, representing a key gap between sequence-level information and observed chromosomal behavior.

Polyploid Triticeae species possess complex genomic constitutions that are traditionally described using subgenome designations derived from their diploid progenitors. For example, bread wheat (*Triticum aestivum*) is a hexaploid species (2n = 6x = 42) composed of three related subgenomes designated A, B, and D, originating from ancestral diploid species of the genera *Triticum* and *Aegilops*. Similar nomenclature is used across the tribe to denote chromosomes originating from different genomes, such as the R genome of rye (*Secale cereale*) or the U and M genomes of several *Aegilops* species. Chromosome arms are conventionally indicated by the letters L (long arm) and S (short arm), and translocations are often described according to the chromosome arms involved (e.g., 4L/5L or 4AL/7BS). These cytogenetic conventions provide a framework for tracing the origin and evolutionary history of chromosomal rearrangements in Triticeae genomes.

## Cytogenetic evidence for repeat-associated chromosome breakpoints

Cytogenetic mapping has long provided compelling evidence that chromosomal rearrangements in the *Triticeae* are non-random and occur preferentially within heterochromatic regions enriched in repetitive DNA [52–55]. Recent studies established that chromosomal breakpoints, translocations, and deletions frequently coincide with loci containing tandem repeats or retrotransposon-rich domains [1, 3, 41, 56]. With the advent of advanced probe systems [11] and high-quality long-read genome assemblies, this relationship has become increasingly evident and mechanistically interpretable.

**Fig. 1:**
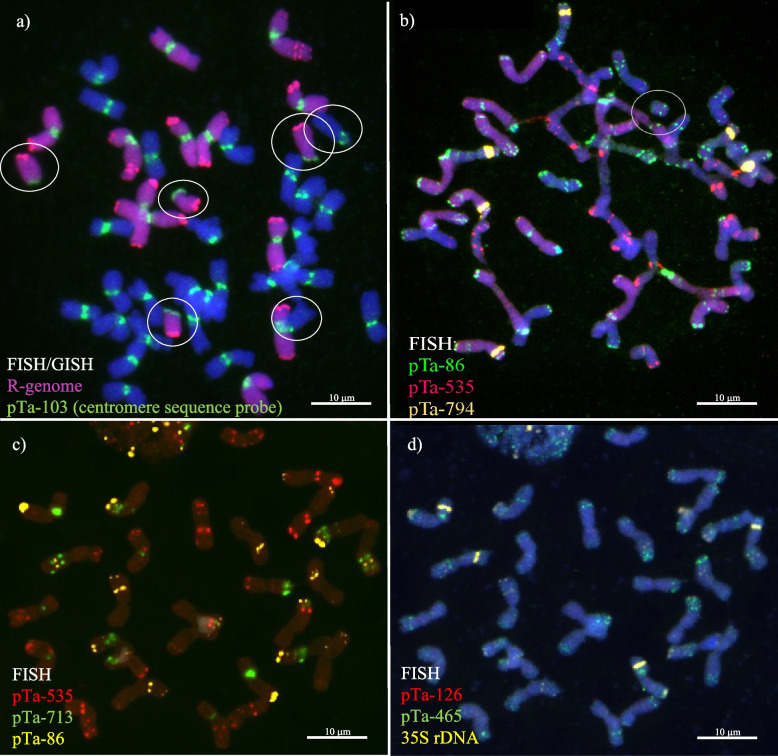
Signals of repetitive sequence probes (pTa-86, pTa-103, pTa-126, pTa-465, pTa-535, pTa-713, 35S rDNA) mapped using fluorescence/genomic in situ hybridization (FISH/GISH) on chromosomes of (**a**) and (**b**) triticale; (**c**) and (**d**) *Triticum turanicum*. Broken chromosomes are circled. Scale bar: 10 μm

**Fig. 2:**
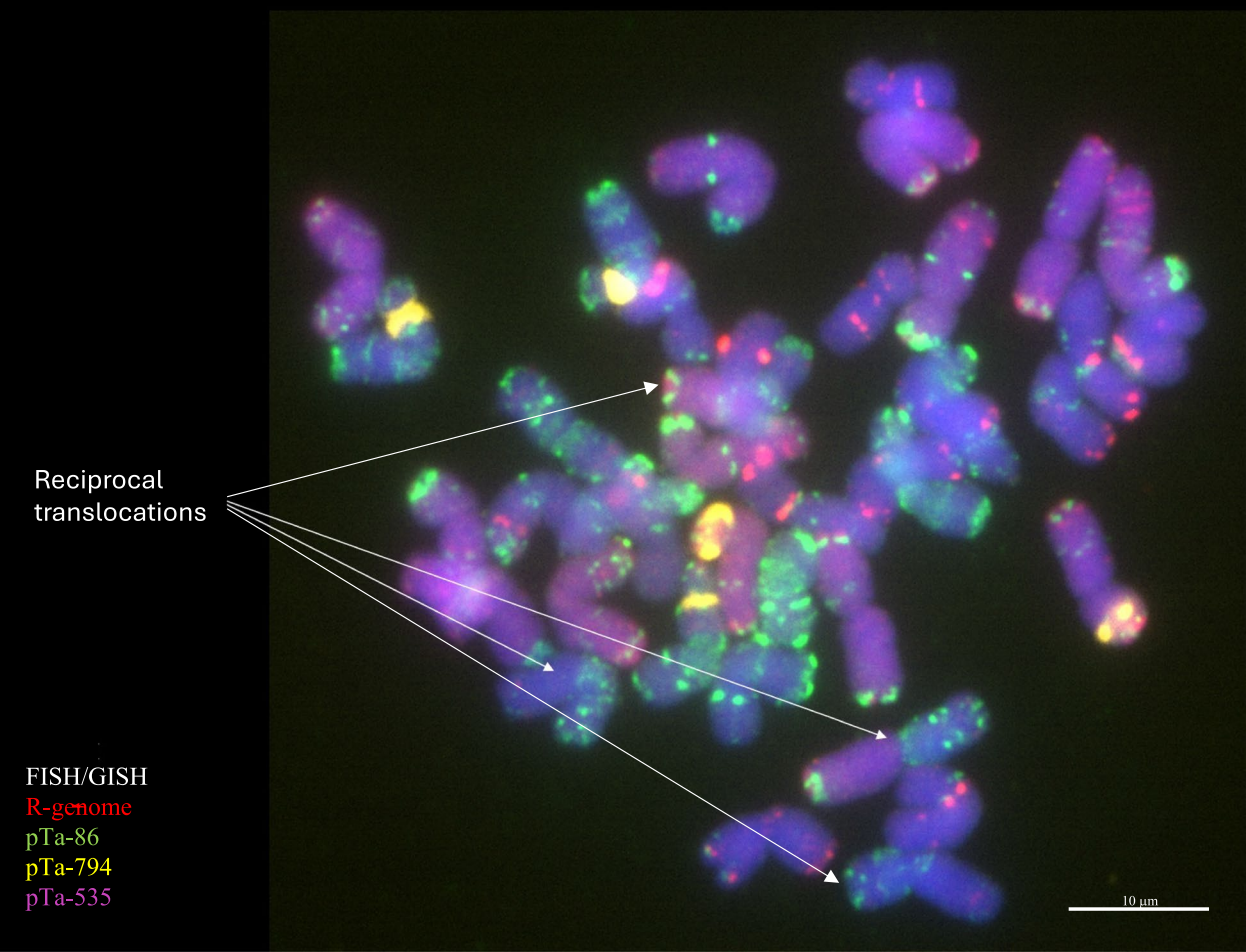
Signals of repetitive sequence probes (pTa-86, pTa-103, pTa-126, pTa-465, pTa-535, pTa-713, 35S rDNA), mapped using fluorescence/genomic in situ hybridization (FISH/GISH) on triticale chromosomes, are indicated by arrows. Reciprocal chromosome translocations are marked by arrows. Scale bar: 10 μm

Early comparative karyotyping using repetitive DNA probes such as pTa-86, pTa-535, pTa-713, and pSc119.2 revealed distinctive hybridization patterns in subtelomeric and pericentromeric regions of *Aegilops*, *Hordeum*, *Secale* and *Triticum* [11, 14, 16, 57, 58] (Figs. 1 and 2). Structural variations—including translocations between rye and wheat chromosomes in triticale and segmental exchanges among the A, B, and D subgenomes of polyploid wheat were consistently localized within or adjacent to these repetitive blocks [13]. Initial GISH experiments further indicated that chromosomal breakpoints often form at junctions where different repeat families overlap, creating structurally unstable interfaces [59].

Recent progress in molecular cytogenetics and genome mapping has provided independent support for the role of repetitive DNA in chromosomal fragility. Burssed et al. demonstrated that structural polymorphisms on chromosomes, manifested as variation in tandem repeat arrays, correlate with recombination frequency and breakpoint localization [1]. Genotypes harboring more complex or polymorphic repeat blocks displayed higher frequencies of chromosomal breakage and non-homologous translocations. Similarly, Tan et al. localized deletion and rearrangement breakpoints in wheat–*Psathyrostachys huashanica* translocation lines to regions enriched in LTR retrotransposons and subtelomeric tandem repeats [60]. Together, these studies confirm that repetitive, heterochromatic regions act as recurrent sites of chromosomal fragility.

A particularly well-characterized example is the 4L/5L translocation (such notation refers to rearrangements involving the long arms (L) of chromosomes 4 and 5) in *Triticum* and *Secale*, which represents a pair of evolutionarily conserved rearrangement hotspots where DSBs have been repeatedly reused [41]. Comparative genomic reconstructions further revealed that several canonical translocations, including 4L/5L and 4AL/7BS, arose independently in different Triticeae lineages and predominantly involve pericentromeric domains enriched in repetitive sequences [19].

Cytogenetic analyses in wheat–barley Robertsonian translocation (RobT) lines provide direct evidence that chromosomal breakage in Triticeae frequently occurs within or adjacent to centromeric regions enriched in repetitive DNA [16]. In *Hordeum vulgare*, approximately 15–20% of cells carrying chromosome 7H exhibited centromeric breaks, most of which were stabilized as telosomes rather than undergoing dicentric fusion [16]. These recurrent centric breakpoints coincide with the positions of tandem and dispersed repeats detected by pAs1 and (GAA)\ₙ probes, supporting the hypothesis that pericentromeric heterochromatin functions as a structural fragility zone within the RDCF framework.

Analyses of *Aegilops tauschii* accession AY61 provided additional insight into the molecular basis of these recurrent rearrangements, identifying multiple, independently formed chromosomal breakpoints involving chromosomes 4, 5, and 7 [5, 19, 41]. When mapped to the ancestral wheat genome, twelve of fourteen identified breakpoints clustered around five chromosomal positions, each reused at least twice during evolution. Two of these correspond to the canonical 4L/5L loci, confirming them as long-term hotspots of chromosomal rearrangement, while the remaining three are located near centromeric regions of chromosomes 4, 5, and 7. These findings provide strong cytogenomic support for the concept of recurrent, sequence-encoded fragility zones within pericentromeric heterochromatin.

Further evidence from independent studies reinforces this model. Small intergenomic translocations during introgression of the *Aegilops longissima Pm13* locus into common wheat, were identified with breakpoints situated within low-complexity, repeat-rich regions [61]. Furthermore, a unidirectional 5AL–7BS translocation in wheat mutants was characterized by dense clusters of repetitive elements surrounding the breakpoint region [62]. Similarly, Danilova et al. analyzed species-specific dispersed repeats in *Aegilops speltoides*, providing comparative insights into chromosomal differentiation that may underlie the recurrent rearrangements observed in interspecific hybrids [24]. At the sequence level, rearrangement hotspots in *A. tauschii* were shown to be enriched in specific retrotransposon families (*Cereba*, *Quinta*) and in regions with high potential for non-B DNA formation [19] supporting the hypothesis that local repetitive architecture modulates fragility and DNA repair dynamics.

Detailed cytogenetic evidence for this phenomenon was presented by Kwiatek et al., who investigated *Aegilops biuncialis* × *Secale cereale* amphiploids crossed with triticale [13]. FISH/GISH analyses demonstrated that intergenomic translocation breakpoints consistently coincided with regions of high repetitive-DNA signal intensity. Recombination events involved triticale chromosomes 1B, 4R, 5R, and 7R (where B denotes a wheat subgenome and R denotes the rye genome), as well as *Aegilops* chromosomes 4U, 5U, 7U, 5 M, 6 M, and 7 M, with breakpoint regions overlapping three major repetitive probe loci. Comparative sequence analysis of these probes (pTa-86, pTa-535, pTa-k566) identified characteristic 8-bp motifs (e.g., TCAATTTC, TCGGTCAT, TTGACCAAT) as signatures of heterochromatic domains associated with chromosome break formation. Comparable rearrangements have been reported in polyploid wheat, where reciprocal arm translocations predominantly involve centromeric regions of chromosomes 4 A, 5 A, 5B, 5D, 6B, 7B, and 2D [52], as well as in triticale, where translocations such as T4RS.4BL, T5BS–5BL.5RL, and T3AS.7RL are localized to repeat-rich heterochromatin [13].

Collectively, these observations indicate that repetitive motifs may function as local modulators of chromosomal fragility. Although their co-occurrence with breakpoints strongly suggests a mechanistic association, their causal role in initiating DSBs or translocations remains hypothetical and requires genome-wide validation. At this stage, the term repetitive fragility zone (RFZ) should be understood not only as a conceptual feature of repeat-rich chromosomes but also as a genomic interval that can be operationally identified. In practical terms, RFZs correspond to chromosomal segments in which repeat architecture coincides with signals of elevated structural instability. Such regions typically show local enrichment of repetitive DNA together with indicators of recurrent rearrangements, including clustering of structural variant breakpoints, the presence of repeat junctions capable of promoting non-allelic homologous recombination, or enrichment of microhomology motifs associated with alternative end-joining repair (Table 2). Because repeat composition and genome organization vary widely across Triticeae species, RFZs are best identified using relative enrichment within chromosomal windows rather than fixed thresholds. In this sense, RFZs represent genomic intervals in which repeat architecture creates a structural context conducive to recurrent chromosome breakage and reorganization.

**Table 2:** Candidate genomic features of repetitive fragility zones (RFZs). References correspond to representative studies illustrating the type of evidence and are not intended to represent an exhaustive list

Feature	Measurable proxy	Evidence type	Status	Example references
High repeat density	proportion of repetitive DNA within genomic window	genome assemblies/repeat annotation	established	[6, 9]
Repeat junction architecture	boundaries between tandem arrays, inverted repeats or direct repeats	sequence architecture analysis	established	[9]
Structural variant breakpoint clustering	local enrichment of structural variant breakpoints	pangenome/long-read SV datasets	established	[5, 27]
Microhomology-rich motifs	enrichment of short homologous motifs near breakpoints	DNA repair pathway analyses	hypothesized	[2, 3]
Centromeric or subtelomeric proximity	chromosomal position relative to centromeres or telomeres	cytogenetic/genome assemblies	established	[16, 19]
Heterochromatic chromatin state	enrichment of heterochromatin marks (e.g. H3K9me2, DNA methylation)	epigenomic datasets	hypothesized	[17, 20, 63]
Late replication timing	replication timing profiles	replication sequencing	hypothesized	[4, 64]
DSB proxy enrichment	SPO11-oligo or γH2AX signals	recombination mapping	hypothesized	[65, 66]
3D chromatin domain boundaries	Hi-C domain edges	chromosome conformation capture	hypothesized	[67–70]

Taken together, cytogenetic and genomic studies consistently show that structural rearrangements in the Triticeae preferentially occur within regions of high repeat density and sequence complexity. The physical co-localization of breakpoints with repetitive loci, observed both cytologically and at the sequence level, supports the view that repetitive DNA is not merely a passive feature of these regions but may contribute to chromosomal instability. The recurrence of these patterns across independent hybrid lineages suggests that chromosomal breakage is unlikely to be purely stochastic and is instead associated with recurrent structural features of these genomic regions. Moreover, the correlation between FISH signal intensity and translocation junctions implies that certain repetitive arrays may influence local chromatin properties, such as torsional stress, condensation, or replication fork progression, and thus may predispose these regions to structural rearrangement.

These observations raise an important question regarding the broader evolutionary scope of this phenomenon within the Triticeae. The tribe encompasses species with diverse genomic constitutions, including the A, B, D, S, H, T, M, and U genomes, which together represent a wide range of repeat compositions and chromosomal architectures. Despite this diversity, comparative cytogenetic and genomic studies suggest that structural rearrangements in many Triticeae species preferentially occur within repeat-rich pericentromeric or subtelomeric regions. The increasing availability of chromosome-scale genome assemblies and pangenome datasets now provides an opportunity to test this hypothesis directly. By comparing breakpoint locations, repeat architectures, and structural variant landscapes across multiple Triticeae genomes, it should be possible to determine whether recurrent rearrangement hotspots correspond to conserved RFZs. Such comparative analyses would allow the proposed RDCF framework to be evaluated across a broader phylogenetic spectrum of Triticeae species.

## Repetitive DNA and meiotic DSB distribution in plants

Meiosis represents a critical process linking genome stability with evolutionary diversification. The controlled induction and repair of DSBs underpin meiotic recombination and ensure faithful chromosome segregation. However, DSB formation is not uniformly distributed across plant genomes; it is strongly influenced by chromatin organization and, in many species, by the density and composition of repetitive DNA. In the large, repeat-rich genomes of the Triticeae, this relationship is particularly pronounced, as most chromatin consists of repetitive sequences that serve as potential substrates for both recombination and structural rearrangement.

Genome-wide mapping of DSBs using DMC1, RAD51, and γH2AX chromatin immunoprecipitation sequencing (ChIP-seq) has revealed distinct distribution patterns among plant species [63, 71]. In *Arabidopsis thaliana* and *Oryza sativa*, DSBs are concentrated in gene-rich, euchromatic regions, whereas pericentromeric and heterochromatic regions exhibit strong suppression of DSB formation. By contrast, large-genome cereals such as maize, barley, and wheat display a more complex landscape: overall DSB density is lower, yet the ratio of DSBs to crossover (CO) events is disproportionately high in repetitive, heterochromatic domains [65, 66, 72]. This suggests that while DSBs can form within repeat-rich regions, their resolution as COs is often suppressed, with repair more frequently proceeding through non-crossover pathways and, in some cases, being associated with structural rearrangements.

In hexaploid wheat, immunolocalization studies have shown that DMC1 and RAD51 foci, cytological markers of DSB sites, extend into interstitial and pericentromeric regions enriched in repetitive DNA, even though CO hotspots remain largely distal and gene-rich [65, 73, 74]. Consequently, wheat heterochromatin has been proposed to behave as “DSB-rich but CO-poor”, a configuration that allows structural reorganization without altering allelic segregation. Although mapping of DSB formation and repair proteins such as RAD51, DMC1, and γH2AX has been performed in model plants, comparable datasets for wheat remain limited. Current evidence supports the view that heterochromatic regions are recombination-poor but structurally dynamic. Rather than being uniformly DSB-rich, these domains may accumulate unrepaired or misrepaired lesions that manifest as chromosomal rearrangements.

The suppression of crossovers within heterochromatin is a conserved eukaryotic feature that preserves chromosome integrity. However, suppression of COs does not imply the absence of DSBs. Heterochromatin regions may accumulate lesions that are repaired through NHEJ or NAHR [75]. In wheat and barley, these pathways appear to dominate within pericentromeric regions, where the abundance and complexity of repetitive sequences hinder homology search. Pangenomic analyses have shown that repeat-rich centromeric and pericentromeric regions harbor exceptionally high densities of structural variants, supporting their role as natural hotspots of chromosomal rearrangement [5].

Repetitive elements influence DSB formation both through intrinsic sequence properties and their effects on chromatin structure. Short motifs with high AT content and palindromic symmetry, attributes associated with DSB hotspots in yeast and mammals [76], may recruit or stabilize SPO11–TOPVIBL complexes responsible for DSB induction, particularly when located within accessible subdomains of heterochromatin. Transposable elements (TEs) frequently create local epigenetic boundaries such as hypomethylated islands within heterochromatin, which can act as weak DSB hotspots due to reduced nucleosome density and increased chromatin accessibility [63, 75]. During meiosis, the expansion of chromatin loops transiently exposes otherwise condensed repetitive regions, increasing the likelihood of replication fork collisions and topoisomerase-mediated cleavage [64].

Another structural feature of meiotic chromosomes that may influence the dynamics of repetitive DNA is the telomere bouquet formed during early prophase I. In many plant species, telomeres attach to the nuclear envelope and transiently cluster within a restricted region of the nucleus, creating a polarized chromosome arrangement that facilitates homologous pairing. Because subtelomeric regions of Triticeae chromosomes are often enriched in tandem repeats and transposable elements, their temporary spatial clustering during the bouquet stage may promote interactions among similar repetitive sequences and potentially influence the evolution of subtelomeric repeat arrays. However, direct evidence linking bouquet formation to structural rearrangements in Triticeae remains limited [77, 78].Together, these factors may help explain why DSB-associated signals tend to cluster at repeat junctions while crossovers remain rare. Most breaks are likely repaired locally or ectopically, consistent with cytogenetic observations of frequent rearrangements in repetitive domains [43, 60].

In polyploid Triticeae, additional complexity arises from the coexistence of homoeologous chromosome sets. The *Ph1* locus in wheat suppresses recombination between homoeologous chromosomes, thereby maintaining diploid-like meiotic behavior. When *Ph1* control is relaxed, as in synthetic or wide hybrids, DSBs within repetitive regions may engage homoeologous sequences, promoting intergenomic exchanges [79–82]. This mechanism explains why translocations in triticale and *Aegilops* × *Secale* amphiploids preferentially occur in repeat-rich heterochromatin, where sequence similarity among the A, B, D, and R genomes provides numerous ectopic recombination partners [43]. Such homoeologous recombination events, initiated by DSBs within repetitive DNA, constitute a major source of chromosomal novelty during early polyploid evolution. Over successive generations, natural selection and epigenetic silencing eliminate deleterious rearrangements, stabilizing only those configurations compatible with essential gene balance—thus reinforcing the fragility–reorganization–stabilization model outlined previously.

The spatial separation between DSB induction and crossover formation creates a genomic “buffer zone” that enables limited experimentation with chromosomal structures without excessive reshuffling of coding regions. Repeat-rich, DSB-prone heterochromatin can therefore be regarded as an evolutionary platform where structural variants originate, are occasionally stabilized, and contribute to genome diversification. This interpretation aligns with comparative evidence showing that major chromosomal rearrangements among wheat subgenomes and related species often trace back to ancient pericentromeric or subtelomeric DSB hotspots [19, 27]. Repetitive DNA thus emerges not only as a challenge to meiotic precision but also as a potential source of evolutionary flexibility, enabling polyploid plants to adapt and differentiate while maintaining essential genome integrity. Moreover, repetitive DNA clusters shape three-dimensional genome architecture by defining heterochromatic domains and compartment boundaries [67]. Although these spatial features correlate with regions of structural variation, direct experimental evidence linking 3D chromatin reorganization to altered DSB formation or chromosomal fragility in wheat is still lacking.

## Molecular mechanisms of repeat-mediated fragility

Chromosomal fragility in repeat-rich regions is thought to arise from a combination of biophysical constraints, replication stress, and DNA repair dynamics that together can convert sequence repetitiveness into structural instability. In the large and repeat-dominated genomes of Triticeae, these processes operate on a genomic scale, linking local molecular events with long-term evolutionary outcomes. Repetitive DNA is inherently capable of forming non-B DNA conformations such as hairpins, cruciforms, triplexes, or slipped-strand structures. These alternative configurations can impede replication fork progression, generate torsional stress, and induce single- or double-strand breaks [46, 76, 83]. Studies in yeast and mammals have demonstrated that short, palindromic, or AT-rich motifs can stall DNA polymerases and serve as substrates for endonuclease-dependent cleavage, thereby promoting DSB formation.

The repair of such breaks can generate diverse classes of structural variants (SVs). In repeat-rich genomes such as those of Triticeae, two principal repair pathways frequently associated with chromosomal rearrangements are NHEJ and NAHR. While NHEJ joins DNA ends with little or no sequence homology and often produces small insertions or deletions, NAHR involves recombination between homologous repeat elements located at different genomic positions and can generate deletions, duplications, inversions, or translocations. In plant genomes, the most common types include (1) deletions, (2) insertions or duplications, (3) inversions, (4) translocations, and (5) complex rearrangements (Table 3). Although these structural changes differ in their genomic outcomes, most originate from the repair of double-strand DNA breaks or replication errors occurring within structurally unstable genomic regions. In repeat-rich genomes such as those of Triticeae, the abundance of homologous or partially homologous sequences strongly influences both the probability of break formation and the repair pathway used.

**Table 3:** Major classes of structural variants (SVs) and their principal molecular formation mechanisms in repeat-rich plant genomes

Structural variant type	Typical molecular mechanism	Characteristic breakpoint signatures	Role of repetitive DNA	References
Deletion	Classical non-homologous end joining (c-NHEJ) or microhomology-mediated end joining (MMEJ) following double-strand breaks	Small insertions or deletions at junctions; short microhomologies	Repetitive elements can promote replication fork stalling or generate closely spaced DSBs, facilitating segment loss	[1, 2]
Insertion/duplication	Replication-based mechanisms such as Fork Stalling and Template Switching (FoSTeS) or Microhomology-Mediated Break-Induced Replication (MMBIR); unequal recombination between repeats	Microhomology at junctions, templated insertions, tandem segment duplication	Tandem repeats or transposable elements provide homologous templates for replication switching	[1, 2]
Inversion	Repair of paired DSBs or recombination between inverted repeats	Breakpoints located within inverted repeat sequences; minimal sequence loss	Inverted repeat arrays facilitate ectopic recombination and orientation reversal of chromosomal segments	[2, 3]
Translocation	Non-homologous end joining between breaks occurring on different chromosomes; ectopic recombination between homoeologous regions	Junctions with minimal homology or short insertions; occasionally microhomology signatures	Sequence similarity among repetitive elements located on different chromosomes facilitates misalignment and interchromosomal exchanges	[1, 3]
Complex rearrangements (chromoanagenesis)	Replication stress and template switching combined with error-prone repair pathways (FoSTeS/MMBIR, alt-EJ)	Clustered breakpoints, mixed fragment orientation, multiple copy-number changes	Repeat-dense regions increase replication instability and template switching, promoting complex genome restructuring	[1, 2]

Deletions represent one of the most common classes of structural variants and frequently arise when DSBs are repaired by classical NHEJ or microhomology-mediated end joining (MMEJ). During these processes, broken DNA ends are rejoined with minimal sequence homology, often leading to the loss of small or large genomic fragments. In repeat-rich regions, replication fork collapse or secondary DNA structures can generate closely spaced breaks whose rejoining results in segmental deletions.

Insertions and duplications often originate through replication-based mechanisms such as FoSTeS or MMBIR. In these cases, stalled replication forks switch templates to nearby homologous or microhomologous sequences, producing segmental duplications or complex insertions. Tandem duplications may also result from unequal recombination between adjacent repetitive elements.

Inversions are typically generated when two breaks occur within a chromosomal segment and the intervening fragment is reinserted in the opposite orientation. In genomes enriched in repetitive DNA, inversions frequently arise through recombination between inverted repeats or through repair of paired DSBs by end-joining pathways.

Translocations involve the exchange of chromosomal segments between non-homologous chromosomes. These rearrangements are often produced when simultaneous breaks occur in different chromosomes and are repaired by NHEJ. In polyploid species or wide hybrids, sequence similarity among homoeologous chromosomes can facilitate ectopic interactions, increasing the probability of interchromosomal exchanges.

Finally, complex rearrangements involving combinations of duplications, deletions, inversions, and translocations may arise during large-scale genome restructuring events collectively described as chromoanagenesis. These include chromothripsis, chromoanasynthesis, and chromoplexy, which involve clustered breakage events followed by error-prone reassembly of chromosome fragments [84]. Replication stress and template switching in repeat-dense regions appear to be important drivers of such complex structural variation.

Together, these mechanisms illustrate how different repair pathways generate distinct classes of structural variants. In repeat-rich genomes such as those of Triticeae, the high density of homologous elements, tandem arrays, and transposable elements increases the likelihood of misalignment and ectopic repair, thereby predisposing specific chromosomal regions to recurrent structural rearrangements.

In plants, replication-fork arrest within heterochromatin activates the ATR-dependent DNA damage response [20, 68]. Because heterochromatic domains often replicate late and are densely compacted, repair protein accessibility may be reduced, potentially increasing the likelihood of incomplete or inaccurate repair. The accumulation of such lesions often results in small deletions, inversions, or translocations. Breaks arising within repetitive regions are typically resolved through NHEJ or NAHR, pathways that favor structural change over precise restoration [26, 75]. NHEJ, predominant in somatic cells, joins DNA ends with minimal or no sequence homology, frequently producing small insertions or deletions. NAHR, in contrast, utilizes sequence similarity between distant repeats, leading to ectopic recombination and exchanges between non-allelic chromosomal segments. These pathway-specific signatures are clearly visible at real chromosomal junctions. Burssed et al. [1] demonstrated that different classes of structural rearrangements exhibit distinct sequence footprints that correspond directly to known DNA repair mechanisms. Simple translocations frequently contain short insertions generated by c-NHEJ, a repair pathway that ligates broken DNA ends with minimal or no sequence homology and often introduces small nucleotide gains or losses. Rearrangements formed via MMEJ or fold-back replication mechanisms display short stretches of microhomology (typically 2–5 bp) at their junctions, reflecting annealing between single-stranded overhangs during end processing. More complex rearrangements, such as those produced by FoSTeS or MMBIR, combine features of both replication stress and template switching, show multiple short microhomologies, inserted fragments derived from nearby templates, and nonlinear segment order. These experimentally characterized breakpoint signatures closely match the expectations of the RDCF model, in which repetitive DNA promotes the formation of DSBs and biases their repair toward non-homologous end-joining, microhomology-mediated annealing, or replication-based template switching—each leaving a distinctive molecular footprint. This mechanism provides a compelling explanation for the frequent intergenomic translocations observed in hybrids and allopolyploids [60]. Given the abundance of highly similar repetitive arrays in Triticeae chromosomes, the probability of misalignment during DSB repair is exceptionally high.

Comparative analyses between *Aegilops tauschii* AY61 and the hexaploid wheat D-subgenome localized individual chromosomal rearrangement (CR) events within 20–200 kb intervals, suggesting that fragility is confined to compact chromosomal domains. Genes flanking these regions display elevated *Ka* and *Ka/Ks* ratios compared with genome-wide averages, consistent with relaxed selective constraints and accelerated evolution near breakpoints [41]. This coupling between sequence instability and local gene evolution underscores both the functional and evolutionary significance of repeat-mediated fragility.

Targeted chromosome engineering in *Arabidopsis thaliana* has shown that large-scale structural rearrangements, including reciprocal translocations between non-homologous chromosomes, can be stably inherited with minimal effects on phenotype, gene expression, or chromatin organization. Despite extensive arm exchanges, translocated lines maintained normal telomere length, histone modification profiles, and transcriptional activity, demonstrating the robustness of higher-order chromatin architecture [56]. These findings indicate that local chromatin context and 3D genome organization may influence the spatial context in which chromosomal breakage and repair occur, although direct mechanistic links remain largely unresolved in Triticeae genomes.

Long-read sequencing has provided direct evidence for repeat-mediated junctions [8, 54, 69], offering molecular confirmation for cytogenetic patterns observed decades earlier. The chromatin environment may influence not only where DSBs form, but also how they are repaired. Heterochromatin is simultaneously protected and fragile: its compact structure may suppress homologous recombination and restrict access of repair factors, potentially allowing DNA lesions to persist. In cereals such as wheat and barley, heterochromatic regions enriched in H3K9me2 and DNA methylation exhibit reduced crossover frequency but elevated DMC1 and RAD51 foci, indicative of frequent DSB formation followed by non-crossover repair [63, 70, 85].

Repetitive elements within centromeric and pericentromeric domains are associated with specialized chromatin states characterized by the centromere-specific histone variant CENH3 and by heterochromatic modifications such as H3K9me2, H3K27me1, and DNA methylation. Paradoxically, this rigidity can increase mechanical stress during chromosomal condensation and segregation. Temporary chromatin relaxation, triggered by environmental stress or polyploidization, may expose repeat-dense regions to DNA damage, initiating rearrangements that are subsequently stabilized through heterochromatinization. Consistent with this view, analysis of the 4L/5L translocation junctions revealed that DSB repair occurred near repeat–gene interfaces, displaying short microhomologies and small insertions characteristic of NHEJ [19]. Detailed sequence studies in *Aegilops tauschii* of recurrent Triticeae breakpoint regions revealed enrichment of centromere-associated retrotransposons such as Cereba and Quinta, which dominate centromeric chromatin in these genomes [8, 19, 22]. These retroelement clusters coincide with local microsynteny disruptions and frequent assembly gaps, reflecting high structural plasticity. The adjacent break regions (BR2 and BR4) show reduced A + T content but increased densities of predicted non-B DNA structures, suggesting that both sequence composition and secondary DNA conformation contribute to fragility. Collectively, these observations indicate that repetitive retrotransposons, compositional bias, and DNA topology act together to generate physically unstable chromosomal interfaces that serve as nucleation points for rearrangement. The centromeric and pericentromeric localization of breakpoints observed in barley [16] parallels the recurrent rearrangement hotspots identified in wheat and *Aegilops*, notably the 4L/5L and 4AL/7BS translocation sites [19, 22]. This convergence across genera underscores the evolutionary conservation of centromere-associated fragility, likely mediated by shared repetitive DNA families such as pAs1 and Ty3/Gypsy retrotransposons.

These findings support the concept that repeat-dense heterochromatin may represent genomic regions prone to recurrent DNA DSBs and structural rearrangements and thus may contribute to genome diversification across Triticeae species. The structural configuration of repetitive chromatin is therefore unlikely to represent a passive substrate for DNA break formation alone, but rather a component of genome architecture that may influence local chromosomal stability. Integrative studies in polyploid wheat have shown that repetitive DNA contributes to the three-dimensional organization of chromatin and may modulate recombination dynamics. High-resolution Hi-C and ChIP-seq analyses [53, 68] indicate that repeat-rich regions form distinct chromatin interaction domains associated with reduced crossover frequency. Within these domains, recombination events appear to be suppressed despite the presence of sequence features potentially conducive to DNA break formation, suggesting a complex balance between chromosomal fragility and structural stability. Alterations in repeat density may also affect chromatin contacts and nuclear organization, indicating that the spatial arrangement of repetitive elements could influence both meiotic behavior and DNA repair dynamics. Consistent with this view, wheat Hi-C studies [67] have shown that repeat clusters contribute to the formation of nuclear interaction domains that appear to act as structural anchors of large-scale genome organization. By bringing distant loci into proximity within three-dimensional chromatin structures, repetitive DNA may increase the likelihood of non-allelic interactions during DNA repair [1]. Although a direct mechanistic link between chromatin conformation and DNA repair selection in wheat remains to be demonstrated, existing evidence supports a model in which sequence composition, chromatin architecture, and repair dynamics jointly influence local chromosomal stability. Although these observations suggest a potential relationship between spatial chromatin organization and the distribution of structural variation, it is important to distinguish between correlation and mechanism. Current evidence in Triticeae primarily derives from comparative analyses indicating that structural variant breakpoints often occur within repeat-rich genomic compartments or near transitions between chromatin domains. However, direct mechanistic evidence demonstrating that three-dimensional genome architecture determines the sites of chromosome breakage or repair partner choice remains limited. In this context, spatial genome organization is best viewed as a structural framework that may influence the probability of interactions between chromosomal loci during DNA repair rather than as a primary driver of genome rearrangements.

Short recurring DNA motifs associated with chromosomal breakpoints in Triticeae [43] indicate that sequence composition contributes to fragility beyond overall repeat abundance. Published analyses of several 8-bp motifs (TCAATTTC, TCGGTCAT, TTGACCAAT) suggest high curvature, AT-richness, and the potential to form hairpin or cruciform structures, features known to enhance susceptibility to nuclease cleavage and replication slippage [1]. These motifs may function as local “fragility seeds,” initiating structural transitions under torsional stress. When embedded within tandem arrays, they can amplify local instability, generating clusters of DSBs observed cytologically as fragile sites or translocation junctions. Recent 3D genomic analyses suggest that the spatial folding of repeat-rich domains imposes additional topological constraints that amplify these effects. Integration of Hi-C data and long-read assemblies across cereal genomes [21] revealed that structural variant (SV) hotspots coincide with spatial boundaries between 3D chromatin domains enriched in transposable elements and satellite repeats. These “domain junctions” have been associated with increased frequencies of DNA breakage and rearrangement, consistent with the RFZs proposed in the present model. The folding of chromatin around repeat blocks may create local topological constraints that predispose these junctions to instability, although a direct mechanistic link between 3D genome architecture and chromosomal rearrangements remains unclear.

From an evolutionary perspective, repeat-driven fragility represents a mechanism of controlled genomic plasticity. Most DSBs are faithfully repaired, but occasional mis-repairs generate structural innovations, translocations, inversions, and duplications, that modify gene regulation and recombination landscapes. Over evolutionary timescales, these rearrangements contribute to speciation, adaptation, and genome size diversification [1].

**Fig. 3:**
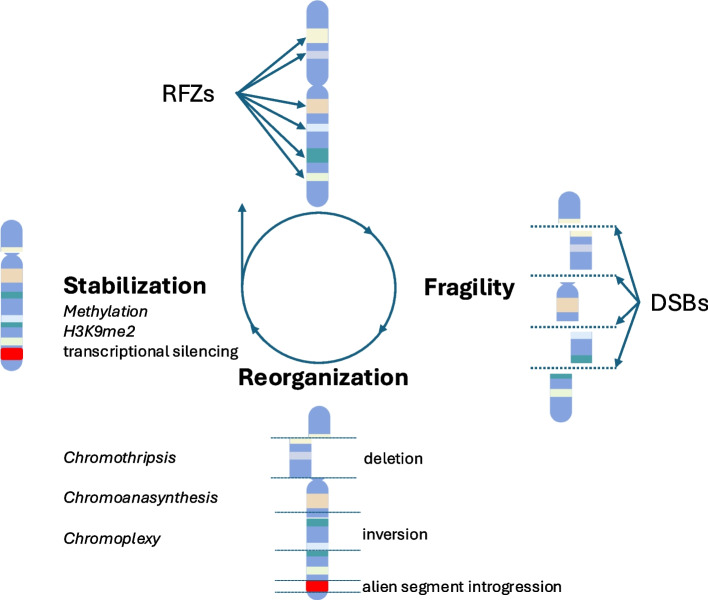
Integration of the repeat-driven chromosomal fragility cycle with chromoanagenesis. The RDCF is depicted as a three-phase cycle. Fragility: repetitive DNA arrays accumulate within heterochromatin and adopt non-B DNA conformations during replication or meiosis, promoting DSB formation at specific loci. Reorganization: error-prone DSB repair and replication-based mechanisms reorganize chromosome structure along three main channels of chromoanagenesis: chromothripsis (clustered shattering and c-NHEJ/MMEJ-mediated re-assembly of chromosome fragments), chromoanasynthesis (FoSTeS/MMBIR-driven DNA segment re-synthesis generating complex copy-number gains), and chromoplexy (multi-chromosomal chains of translocations formed by c-NHEJ/alt-EJ). Stabilization: the resulting derivative chromosomes become epigenetically sealed through heterochromatin formation (e.g. H3K9me2, DNA methylation) and transcriptional silencing. Repeat accumulation within these newly formed heterochromatic blocks re-initiates fragility, closing the cycle and linking repeat dynamics to recurrent chromoanagenesis events

At the genomic scale, this process follows a three-phase cycle (Fig. 3):

1. *Fragility* — RFZs are proposed to represent genomic regions predisposed to DSB formation at specific sites within heterochromatin.
2. *Reorganization* — encompasses the diverse error-prone pathways that repair these breaks or restart disrupted replication forks. Depending on the cellular context, this reorganization proceeds along three principal routes of chromoanagenesis [84]:
  - Chromothripsis—in which a chromosome undergoes extensive fragmentation followed by chaotic reassembly via classical NHEJ or MMEJ.
  - Chromoanasynthesis—driven by FoSTeS or MMBIR, which generates complex combinations of duplications, triplications, and inversions during aberrant DNA resynthesis.
  - Chromoplexy -where DSBs arising simultaneously in multiple chromosomes are rejoined into chains of interchromosomal translocations through c-NHEJ or alt-EJ.
3. *Stabilization* — the resulting chromosomal configurations become epigenetically sealed through heterochromatinization, involving, for example H3K9me2 and DNA methylation, and transcriptional silencing, after which new repeats may accumulate, restarting the cycle.

This dynamic loop explains why repeat-rich regions function as long-term engines of structural and evolutionary change. Through alternating phases of instability and fixation, the genome evolves in a stepwise, non-random manner. The fragility–reorganization–stabilization model thus reconciles the dual nature of repetitive DNA—as both a source of instability and a scaffold for new genomic states. This perspective echoes Barbara McClintock’s concept of the genome as a “responsive system” [86], now reinterpreted through the lens of modern genomics. In Triticeae, where polyploidization and interspecific hybridization are recurrent, repeat-driven reorganizations likely underlie the extensive karyotypic diversity observed across the tribe. Repetitive DNA and its associated motifs therefore emerge not as inert remnants of genome expansion but as active evolutionary agents driving both instability and innovation in plant genomes.

## Conceptual scope and key predictions of the RDCF framework

The molecular mechanisms discussed above, including NAHR, replication stress associated with repetitive DNA, formation of non-B DNA structures, and error-prone end-joining repair pathways, are well established drivers of structural variation across many eukaryotic genomes. The RDCF framework proposed here does not introduce a new molecular mechanism of DNA breakage or repair. Instead, it provides an integrative conceptual model explaining why these processes repeatedly generate chromosomal rearrangements at specific genomic locations in Triticeae genomes. In this framework, the central determinant is the local architecture of repetitive DNA. Regions containing dense clusters of tandem repeats, transposable elements, and short sequence motifs may create structurally unstable chromatin environments prone to replication stress and DSB formation. When such regions repeatedly act as substrates for different DNA repair pathways, they may form recurrent breakpoint regions referred to here as RFZs. The RDCF model therefore links three levels of genome organization (repeat architecture, chromatin context, and recurrent rearrangements) within the fragility–reorganization–stabilization cycle described above (Fig. 3).

If the RDCF model correctly describes the relationship between repetitive DNA architecture and chromosomal instability in Triticeae genomes, several testable predictions follow. First, structural variant breakpoints should be enriched within repeat-dense genomic regions relative to the genomic background. Second, recurrent rearrangements observed in different Triticeae species or hybrid lineages should preferentially involve the same repeat-rich chromosomal domains. Third, RFZs should correspond to regions characterized by high repeat density, reduced recombination, and increased susceptibility to replication stress. Fourth, comparative analyses of chromosome-scale genome assemblies should reveal that many breakpoint regions coincide with conserved repeat clusters or repeat junctions across related Triticeae genomes. Together, these predictions provide a framework for testing the proposed model using comparative genomics, structural variant mapping, and cytogenetic approaches.

Alternative explanations should also be acknowledged. Some rearrangements may reflect general replication stress, chromatin reorganization during polyploidization, or selective retention of viable breakpoint configurations. The RDCF framework therefore does not replace established molecular mechanisms of structural variation but proposes that in Triticeae repetitive DNA architecture is a major factor shaping the positional recurrence of chromosomal breakpoints.

## Concluding remarks and perspectives

Repetitive DNA, once often considered functionally neutral genomic material, is now recognized as an important component of genome architecture and evolution. The ancestral genome reconstructions in Triticeae now enable precise mapping of recurrent breakpoints and their sequence composition [19]. Integrating such phylogenetically anchored structural data with cytogenetic and motif-based analyses could help to test whether recurrent chromosomal rearrangements preferentially arise at predicted fragile motifs within heterochromatic domains. Across Triticeae and many other plant groups, evidence from cytogenetic, genomic, and epigenetic studies converges on a consistent theme: chromosome breakpoints, translocations, and other structural variations arise preferentially within repeat-rich heterochromatic regions. These domains are not merely passive targets of instability, they may also contribute to its emergence. Building upon decades of cytogenetic observations and supported by recent advances in high-resolution genomics, this review proposes a unified framework of RDCF. According to this model, short repetitive motifs may serve as molecular signatures of fragility, marking local domains prone to DSBs. Recent fine-scale analyses of Triticeae genomes have identified a limited number of recurrent chromosomal breakpoint regions clustering in repeat-rich pericentromeric domains. The recurrence of these sites across independently evolved Triticeae lineages suggests that fragility may be, at least in part, predictable from sequence composition and chromatin context. Integrating such empirical data with the RDCF framework offers a practical path toward quantitative “fragility maps” of cereal genomes. When embedded within compact heterochromatin, such motifs promote the formation of non-B DNA structures, replication stress, and erroneous repair, ultimately generating the recurrent structural rearrangements that shape both hybrid and polyploid lineages of Triticeae.

Through the fragility–reorganization–stabilization cycle described in the RDCF framework (Fig. 3), repeat-driven processes may continuously remodel genome architecture. Each cycle leaves behind rearranged and epigenetically sealed chromosomal configurations, which over evolutionary time become substrates for further innovation. In this way, genomic instability and genome evolution may be viewed not as opposing forces, but as closely related consequences of repetitive genome architecture. This dynamic equilibrium explains how polyploid plants reconcile immense genome size and redundancy with remarkable evolutionary flexibility. Comparable mechanisms have been identified in *Brassica*, *Brachypodium*, *Solanum*, and even in fungi and animals, suggesting that repetitive DNA–mediated fragility may represent a broadly relevant evolutionary principle. It provides the raw material for structural variation, drives karyotype divergence, and underlies both adaptation and speciation in complex genomes.

Looking forward, integrating motif discovery, DSB mapping, and chromatin profiling will make it possible to develop predictive “fragility maps” for crop genomes—tools capable of identifying regions prone to structural rearrangements. Such maps could transform plant breeding and genome engineering, allowing not only the avoidance of unwanted instability but also the strategic use of controlled fragility to facilitate beneficial gene exchanges and introgressions. Ultimately, the RDCF framework unites molecular sequence features, chromatin biology, and evolutionary theory into a single paradigm. It reveals that the same repetitive sequences once considered genomic debris are, in fact, important contributors to genetic innovation, diversity, and adaptability in plant genomes. In the vast and repeat-dominated genomes of the Triticeae, fragility may be viewed not simply as a liability, but as an important contributor to their complexity and resilience. DNA repetitive motifs may act as modulators of chromosome fragility in cereals, particularly in repeat-dense regions, but their universality and mechanistic role remain to be established through comparative and functional analyses.

Despite the revolutionary potential of modern sequencing and gene-editing technologies, the biological reality of complex, polyploid plant genomes continues to defy full predictability and control. Even as CRISPR and synthetic genomics expand the precision of targeted edits [56], the creation of novel allelic combinations and adaptive chromosomal architectures will continue to rely on natural mechanisms of recombination and hybridization**.** In this context, the classical principles of cytogenetics regain contemporary relevance: understanding where and why chromosomes break and rejoin is fundamental to harnessing genetic variation without compromising genome stability. Conventional crossing and the exploitation of wild relatives will remain indispensable strategies for introducing complex traits such as stress tolerance, yield stability, and fertility restoration. Yet their success depends critically on the cytogenetic stability of hybrids and introgression lines**,** which in turn is determined by the distribution of fragile and translocation-prone chromosomal regions. The RDCF model therefore provides both a conceptual and practical bridge between classical breeding and modern genomics.

By integrating molecular, epigenetic, and structural insights, we can move toward predictive maps that help breeders anticipate chromosomal behavior in hybrid backgrounds. The future of plant improvement will rest not solely on the precision of gene editing, but on the depth of our understanding of how genomes naturally rearrange and evolve**.** In the long term, embracing and harnessing chromosomal fragility**,** rather than eliminating it, may prove to be the key to sustaining the evolutionary creativity that has driven crop diversity for millennia.

## Data Availability

The datasets used and analyzed during the current study are available from the corresponding author on reasonable request.

## General genomic and cytogenetic terms

3D: Three-dimensional
ChIP-seq: Chromatin Immunoprecipitation Sequencing
CO: Crossover
DSB: Double-strand break
FISH: Fluorescence in situ hybridization
GISH: Genomic in situ hybridization
Hi-C: Chromosome conformation capture sequencing
LCR: Low-copy repeat
LTR: Long terminal repeat
RFZ: Repetitive Fragility Zone
RDCF: Repeat-Driven Chromosomal Fragility
SV: Structural variant
TE: Transposable element

## Meiotic and DNA repair proteins and pathways

ATR: Ataxia Telangiectasia and Rad3-related protein
alt-EJ: Alternative end-joining
c-NHEJ: Classical non-homologous end joining
DSBR: Double-strand break repair
FoSTeS: Fork Stalling and Template Switching
MMBIR: Microhomology-Mediated Break-Induced Replication
MMEJ: Microhomology-mediated end joining
NAHR: Non-allelic homologous recombination
NHEJ: Non-homologous end joining
RAD51: DNA repair protein RAD51
SPO11: Topoisomerase-like protein introducing meiotic DSBs
TOPVIBL: Topoisomerase VIA binding protein, SPO11 partner

## Chromoanagenesis mechanisms

RDCF: Repeat-Driven Chromosomal Fragility
Chromothripsis: Chromosome shattering and random reassembly
Chromoanasynthesis: Replication-based complex rearrangement
Chromoplexy: Multichromosome DSB repair leading to chained translocations

## Chromatin and epigenetic marks

H3K9me2: Histone H3 lysine 9 dimethylation
H3K27me3: Histone H3 lysine 27 trimethylation
γH2AX: Phosphorylated H2AX, DSB marker
CENH3: Centromere-specific histone H3 variant
H3K27me1: Histone H3 lysine 27 monomethylation

## Repetitive DNA markers used in FISH

pTa-86: Wheat tandem repeat probe
pTa-103: Wheat repetitive sequence probe
pTa-126: Wheat repetitive sequence probe
pTa-465: Wheat tandem repeat probe
pTa-535: Wheat repetitive sequence probe
pTa-713: Wheat repetitive sequence probe
pSc119.2: Rye subtelomeric tandem repeat
pAs1: Aegilops tandem repeat
35S rDNA: Ribosomal DNA repeat (nucleolar organizer)

## Genomic elements/repeat families

Ty1/Copia: LTR retrotransposon superfamily
Ty3/Gypsy: LTR retrotransposon superfamily
Cereba: Centromeric retrotransposon family
Quinta: Retrotransposon family in Triticeae

## Species and genome terms

A, B, D genomes: Three subgenomes of hexaploid wheat
*Ph1*: Pairing homoeologous 1 locus controlling recombination in wheat

## Specific chromosome introgression notations

7NsS: Short arm of chromosome 7 from *Psathyrostachys huashanica*
5AL, 7BS, etc.: Wheat chromosomes (long/short arms indicated by L/S)
